# Predicting clinical events using Bayesian multivariate linear mixed models with application to scleroderma

**DOI:** 10.1186/s12874-021-01439-y

**Published:** 2021-11-14

**Authors:** Ji Soo Kim, Ami A. Shah, Laura K. Hummers, Scott L. Zeger

**Affiliations:** 1 Department of Biostatistics, Johns Hopkins Bloomberg School of Public Health, Baltimore, MD USA; 2 Division of Rheumatology, Department of Medicine, Johns Hopkins University School of Medicine, Baltimore, MD USA

**Keywords:** Bayesian hierarchical models, Longitudinal profiles, Multivariate mixed models, Sequentially-updated prediction, Scleroderma

## Abstract

**Background:**

Scleroderma is a serious chronic autoimmune disease in which a patient’s disease state manifests in several irregularly spaced longitudinal measures of lung, heart, skin, and other organ systems. Threshold crossings of pulmonary and cardiac measures indicate potentially life-threatening key clinical events including interstitial lung disease (ILD), cardiomyopathy, and pulmonary hypertension (PH). The statistical challenge is to accurately and precisely predict these events by using all of the clinical history for the patient at hand and for a reference population of patients.

**Methods:**

We use a Bayesian mixed model approach to simultaneously characterize each individual’s future trajectories for several biomarkers. We estimate this model using a large population of patients from the Johns Hopkins Scleroderma Center Research Registry. The joint probabilities of critical lung and heart events are then calculated as a byproduct of the mixed model.

**Results:**

The performance of this approach is substantially better than standard, more common alternatives. In order to predict an individual’s risks in a clinical setting, we also develop a cross-validated, sequential prediction (CVSP) algorithm. As additional data are observed during a patient’s visit, the algorithm sequentially produces updated predictions for the future longitudinal trajectories and for ILD, cardiomyopathy, and PH. The updated prediction distributions with little additional computing, for example within an electronic health record (EHR).

**Conclusions:**

This method that generates real-time personalized risk estimates has been implemented within the electronic health record system for clinical testing. To our knowledge, this work represents the first approach to compute personalized risk estimates for multiple scleroderma complications.

**Supplementary Information:**

The online version contains supplementary material available at 10.1186/s12874-021-01439-y.

## Background

It is a major challenge to assess risks of critical events in chronic, multi-organ diseases such as multiple sclerosis [1], lupus [2], and Parkinson’s disease [3]. Scleroderma is an autoimmune disease that causes excessive fibrosis, vasculopathy and immunological derangements that can affect multiple organ systems including the skin, heart, lungs, kidney, gastrointestinal tract, muscles, joints, and blood vessels. The hallmark of scleroderma is its significant heterogeneity and variable risk of internal organ involvement across patients. Severe organ involvement can result in early death, and there is a critical unmet need to identify patients at high risk of progression at an early stage of the disease [4, 5]. The 9-year cumulative survival rate for diffuse scleroderma patients with severe organ involvement was estimated to be 38% [5]. Mortality is highest due to pulmonary and cardiac complications of the disease; 35% of scleroderma-related death has been attributed to pulmonary fibrosis, 26% to pulmonary arterial hypertension (PAH) and 26% to cardiac causes [6]. Such events are commonly observed in scleroderma patients; for example, pulmonary involvement has been reported in up to 25% of patients at the early stage of diagnosis [7]. Hence, a major clinical goal at an early stage of the disease is to identify patients who are most likely to progress, as this may provide a window of opportunity to intervene before there is irreversible organ damage [8].

In monitoring scleroderma, clinicians obtain serial pulmonary function tests and echocardiograms to screen for emerging cardiac and pulmonary complications. Left ventricular ejection fraction (EF), right ventricular systolic pressure (RVSP), and percent predicted forced vital capacity (pFVC) are monitored to detect whether there is emerging cardiomyopathy, pulmonary hypertension (PH) and interstitial lung disease (ILD), respectively. For each of these measures, a value above or below clinically established thresholds is a surrogate for these endpoints.

Numerous statistical methods have been developed to quantify the risk of a major clinical event associated with chronic diseases. Ky et al. used time-dependent Cox regression to predict heart failure [9]; the Emerging Risk Factors Collaboration used a similar approach to predict cardiovascular events from novel biomarkers [10]. Nelson et al. used pooled hazard regression models from 34 cohorts to predict the risk of incident chronic kidney disease [11]. Machine learning approaches have also been used, for example to predict sepsis among ICU patients [12] and onset of any major clinical event for patients in the wards [13].

In this study, the events of interest are defined by the values of continuous biomarkers crossing a threshold. Hence, by estimating the joint distribution of the biomarkers, we can obtain accurate and precise predictions of multiple major scleroderma events.

If the events are discrete events rather than threshold crossings, for example death or renal crisis in scleroderma patients, joint models of repeated biomarker measures and times-to-events have been developed. The joint model proposed by Faucett and Thomas, 1996 [14] and Wulfsohn and Tsiatis, 1997 [15] are early examples. Several extensions were proposed to accommodate multivariate longitudinal profiles. Xu and Zeger, 2001 [16] used a multivariate mixed model framework to model multiple continuous surrogate markers to evaluate treatment effect in a schizophrenia trial and proposed a measure to quantify the relative benefits of using multiple surrogates. Rizopoulos and Ghosh, 2011 [17] propose a semiparametric multivariate joint model to model three longitudinal outcomes and time to renal graft failure. Other applications are presented by Brown, Ibrahim, and DeGruttola, 2005 [18], Proust-Lima and Taylor, 2009 [19], and Garre et al., 2008 [20]. This literature is summarized in books by Rizopoulos and Elashoff et al., 2012 [21] and 2016 [22].

While there is a rich literature on modeling continuous and discrete longitudinal biomarkers, prediction of future binary events is almost always done using Cox or logistic regression or more recently, machine learning algorithms as referenced above. In this currently favored approach, simple summaries of the biomarker histories, for example the last value or recent trend, must be selected thereby setting aside the rest of the information in the biomarker process. Biomarker measurement error, irregular observation times, and missing values are challenges in these prediction models. With lung and heart function in this study, and with many other important disease outcomes, for example hypertension, obesity, end stage renal disease, and diabetes, the key events are defined to be known functions of one or more of the biomarkers. If such cases, more information is preserved by modeling the joint biomarker process and then deriving the distribution of the events from the biomarker model rather than directly modeling the events. Because most biomarker processes are not Gaussian, the biomarker models must flexibly adapt to differently-shaped marginal distributions.

In this paper, we use a flexible statistical model for a set of scleroderma biomarkers to predict major clinical events and compare this prediction strategy to the more common direct modeling of the events. We use linear mixed effects models for the multivariate longitudinal outcomes and their dependence on clinically-relevant predictor variables. This approach allows each individual to have a unique trajectory and quantifies the degree of heterogeneity among individuals. We estimate model parameters and individual trajectories using a Bayesian approach implemented using Markov chain Monte Carlo for a large population of scleroderma patients from a major academic health center. To quantify an individual’s risk, the joint posterior distribution of the critical lung and heart events is calculated from the estimated model parameters. The relative performance of this multivariate longitudinal approach is compared to common alternatives for binary outcomes including multiple logistic regressions and random forest machine learning algorithms.

The standard multivariate linear mixed effects model assumes that the response variables are approximately jointly Gaussian, which is not the case in our application and many others. Therefore, we preprocess the biomarker data by applying a non-parametric transformation to both the outcomes and the thresholds that define the events. While multivariate linear mixed effects models are in common use, their estimation is computationally intensive and therefore not amenable for clinical use. Hence, we also develop a cross-validated, sequential prediction algorithm (CVSP) for multivariate longitudinal data that combines the outputs from prior model runs with new data for the patient at hand.

In the following sections, we provide a brief overview of the Bayesian multivariate linear mixed effects model and its application to predicting binary events defined by threshold crossings. We specify the preprocessing method that extends the application of our approach to non-Gaussian biomarker distributions. We provide details of the Bayesian multivariate mixed model fitted to the transformed data to estimate individuals’ risks of the critical events. We then present details of our novel algorithm for producing a patient’s risk of future events from their updated data without having to refit the models. We compare the performance of our prediction approach to a series of models that use the past events themselves along with biomarker trajectories as the main predictors of future events. The discussion addresses the important steps statisticians must take to have a prediction method like this one contribute to clinical practice.

## Methods

### Modeling multivariate measures and events

**Fig. 1:**
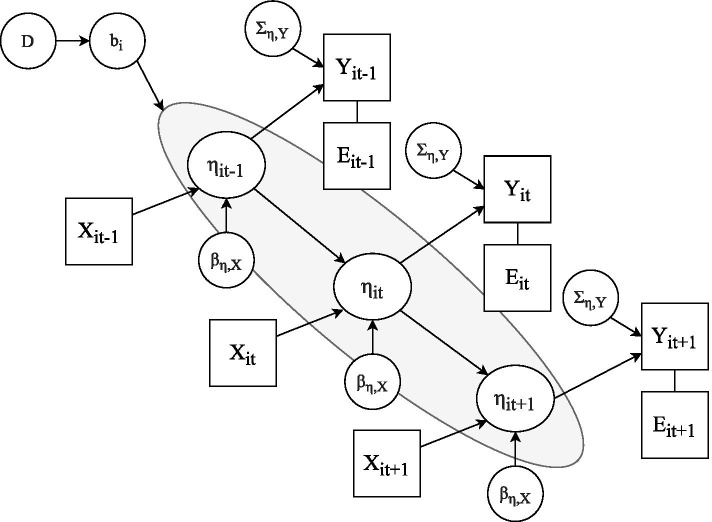
The latent disease state is *η*_*is*_ that causes observations comprising a *K*_*Y*_ -vector of biomarkers *Y*_*it*_ and a binary *K*_*E*_ vector of event indicators *E*_*it*_ at observation times ${t}_i=\left({t}_{i1},{t}_{i2},\dots, {t}_{i{n}_i}\right)$. *η*_*is*_ is affected by interventions and other covariates *X*_*it*_ through regression coefficients *βη*,_*X*_ and by zero-mean random effects *b*_*i*_ with covariance matrix *D*. The conditional distribution of the biomarkers given disease state is assumed to be Gaussian with mean *X*_*it*_*β*_*η*, *X*_ and covariance matrix Σ_*η*, *Y*_

Motivated by the scleroderma example, we develop and apply a Bayesian multivariate mixed effects model to estimate the conditional distribution of each patient’s future biomarker trajectories and even risks given her clinical history. To establish notation, Fig. 1 represents the key assumptions as a directed acyclic graph (DAG) in which variables are nodes and causal relationships are directed edges. Variables in ovals are unknowns; those in squares are observations. The latent disease state for patient *i* with *n*_*i*_ observations is denoted *η*_*is*_. It is manifested in a vector of *K*_*Y*_ biomarkers *Y*_*it*_ and events *E*_*it*_ at observation times ${t}_i=\left({t}_{i1},{t}_{i2},\dots, {t}_{i{n}_i}\right)$. The disease state is affected by interventions and other covariates *X*_*it*_ through regression coefficients *β*_*η*, *X*_ and by random effects *b*_*i*_ with mean 0 and covariance matrix *D*. The conditional distribution of the biomarkers *Y*_*it*_ given disease state *η*_*it*_ is assumed to be Gaussian with covariance matrix *Σ*_*η*,*Y*_. The binary vector of events *E*_*it*_ are deterministic functions of *Y*_*it*_ indicating whether or not biomarkers crossed known thresholds.

We represent the complex disease state involving multiple organs by jointly fitting all biomarkers in a single model. Univariate analyses in which each outcome measure or event is considered on its own are more popular largely because modeling is simpler. However, in such models, the across-measure associations in the random effects and residual errors, captured by off-diagonal elements of *D* and *Σ*_*η*,*Y*_, are ignored. Failure to account for these associations results in less efficient estimators of the fixed and random effects [23–28].

### Multivariate outcome model

Longitudinal biomarkers from the Johns Hopkins Scleroderma Center Research Registry include: ejection fraction (EF), right ventricular systolic pressure (RVSP), percent predicted forced vital capacity (pFVC), and percent predicted diffusing capacity of carbon monoxide (pDLCO). These measures are used to describe the disease trajectory of patients who have at least two observations for each of the measures. We use the earlier date of the onset of Raynaud’s phenomenon and first non-Raynaud’s symptom as the patient’s onset of disease (*t* = 0). We restrict our analysis to data collected between 0 to 40 years since onset. Clinicians define thresholds for EF, RVSP, and pFVC events below or above which the patient is said to experience: cardiomyopathy, PH, and ILD, respectively. Note that these events can occur multiple times for each patient. We use two thresholds for each measure to differentiate between mild and severe events as shown in Table 1.

**Table 1:**
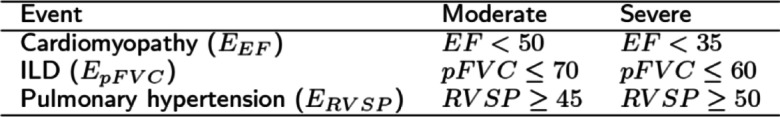
Mild and severe clinical events defined by thresholds

**Fig. 2:**
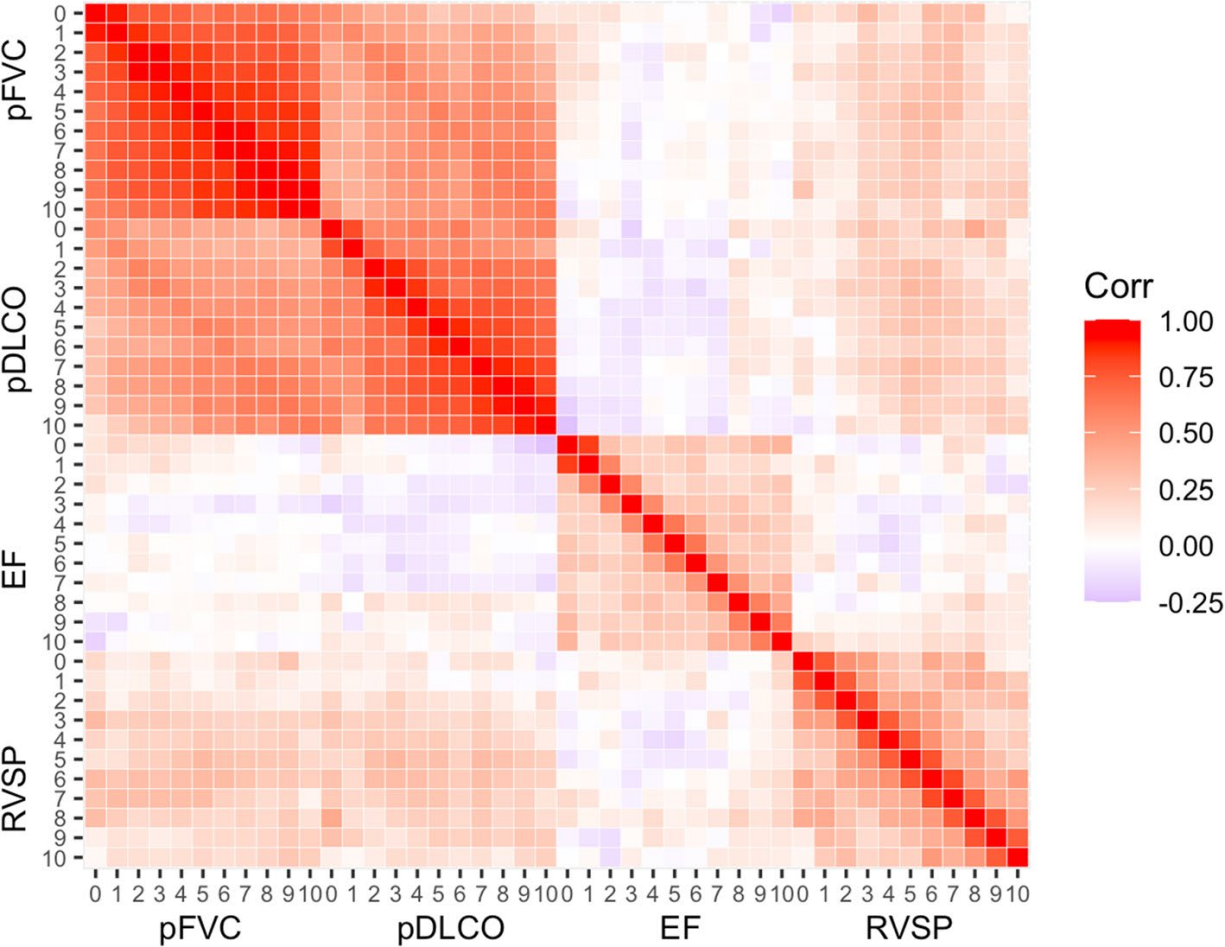
Empirical correlation matrix of the pre-processed variables. Pairwise correlations of observations from all patients for 11 years (years 0, …,10 since the disease onset) are calculated and plotted using range of colors from red, white, and blue each representing correlation of 1, 0, and -1, respectively. The 11 by 11 block matrices on the diagonals show the degree of correlation in patients’ repeated observations over time for each of the four measures, and the 11 by 11 block matrices of the diagonal display the degree of correlation across different measures

We have a choice of building models with any combination of three multivariate outcomes EF, RVSP, and pFVC to obtain predictions for *E*_*EF*_, *E*_*RVSP*_, and *E*_*pFVC*_. Along with the three measures, we chose to include pDLCO in the model as the two lung measures pFVC and pDLCO are known to be highly correlated, as shown in the empirical correlation matrices in Fig. 2. We also observe that RVSP observations are highly correlated with pDLCO and pFVC across time. We fit a multivariate linear mixed model with four longitudinal outcomes: pFVC, pDLCO, EF, and RVSP. For any patient at a given moment in the future, we can obtain *p*(*E*_*EF*_), *p*(*E*_*FVC*_), and *p*(*E*_*RVSP*_) from the model.

### Preprocessing of longitudinal data

Prior to analysis, all 4 outcome measures are pre-processed using quantile normalization to make their marginal distributions more nearly Gaussian. Let *k* = 1, …, 4 denote measures pFVC, pDLCO, EF, and RVSP, and let *Y*_*k*_ be a vector of the observed values from each measure *k*. The quantile normalized vector is obtained by ${\Phi}^{-1}\circ {\hat{G}}_k\left({Y}_k\right)$ where ${\hat{G}}_k$ is an estimated marginal distribution function of the vector *Y*_*k*_ and Φ^−1^ is the inverse of the standard Gaussian distribution. Quantile normalization is widely used in the analysis of microarray data [29] and other areas of application [30–32].

Thresholds for the three events are also transformed to the normalized scale, which we call *c*_*EF*_, *c*_*RVSP*_, and *c*_*pFVC*_. Note that transforming each measure individually does not guarantee their joint Gaussianity of the random errors and random effects. Below we propose a simple method to check whether the joint Gaussian assumption is seriously violated.

### The multilevel response models and prediction

Let *y*_*ijk*_ be the observed value for the *k*th measure for person *i* = 1, …, *m* at the *j* th visit *j* = 1, …, *n*_*ik*_, at time *t*_*ijk*_. Let *Y*_*ik*_ be the vector of *y*_*ijk*_ for *j* = 1, …, *n*_*ik*_. Define *X*_*ik*_ and *Z*_*ik*_ to be (*n*_*ik*_ × *p*_*k*_) and (*n*_*ik*_ × *q*_*k*_) matrices of the predictors for fixed and random effects, respectively. Let *β*_*k*_ and *b*_*ik*_ are (*p*_*k*_ × 1) and (*q*_*k*_ × 1) measure-specific vectors of fixed and random effects regression coefficients. Let ${n}_i=\sum_{k=1}^K{n}_{ik}$ and *e*_*ik*_ random measure-specific within-subject error term.

The linear mixed effects model is written as *Y*_*i*_ = *X*_*i*_*β* + *Z*_*i*_*b*_*i*_ + *e*_*i*_, *i* = 1, …, *m* and $\beta ={\left({\beta}_1^T,\dots, {\beta}_K^T\right)}^T,{Y}_i={\left({Y}_{i1}^T,\dots, {Y}_{iK}^T\right)}^T$,

$${X}_i=\left(\begin{array}{cccc}{X}_{i1}& \mathbf{0}& \cdots & \mathbf{0}\\ {}\mathbf{0}& {X}_{i2}&\ & \vdots \\ {}\vdots &\ & \ddots & \mathbf{0}\\ {}\mathbf{0}& \cdots & \mathbf{0}& {X}_{iK}\end{array}\right),{Z}_i=\left(\begin{array}{cccc}{Z}_{i1}& \mathbf{0}& \cdots & \mathbf{0}\\ {}\mathbf{0}& {Z}_{i2}&\ & \vdots \\ {}\vdots &\ & \ddots & \mathbf{0}\\ {}\mathbf{0}& \cdots & \mathbf{0}& {Z}_{iK}\end{array}\right),$$

where **0** is a matrix of zeros. We assume ${b}_i={\left({b}_{i1}^T,\dots, {b}_{iK}^T\right)}^T\ {\sim}^{ind}\ {N}_{Kq}\left(0,D\right)$ and ${e}_i={\left({e}_{i1}^T,\dots, {e}_{iK}^T\right)}^T{\sim}^{ind}\ {N}_{n_i}\left(0,{\Sigma}_{\mathrm{i}}\right).$

Now, consider ${Y}_{i{j}^{+}}$, the *K* × 1 vector of patient *i*’s health state at an unobserved future time ${t}_{i{j}^{+}}$, and its predictor matrices ${X}_{i{j}^{+}}$ and ${Z}_{i{j}^{+}}$. To predict the probability of clinical events at ${t}_{i{j}^{+}}$, we use the conditional distribution of ${Y}_{i{j}^{+}}$, given *Y*_*i*_, the vector of observations prior to ${t}_{i{j}^{+}}$. The joint distribution of the history of observations *Y*_*i*_ and a future observation ${Y}_{i{j}^{+}}$, is

$$\left(\begin{array}{c}{Y}_i\\ {}{Y}_{i{j}^{+}}\end{array}\right)\sim N\left(\left(\begin{array}{c}{X}_i\beta \\ {}{X}_{i{j}^{+}}\beta \end{array}\right),\left(\begin{array}{cc}{V}_i& {C}_{i{j}^{+}}\\ {}{C}_{i{j}^{+}}^T& {V}_{i{j}^{+}}\end{array}\right)\right)$$

where ${V}_i={Z}_iD{Z}_i^T+{\Sigma}_i,{V}_{i{j}^{+}}={Z}_{i{j}^{+}}D{Z}_{i{j}^{+}}^T+{\Sigma}_{i{j}^{+}}$, and ${C}_{i{j}^{+}}={Z}_iD{Z}_{i{j}^{+}}^T,{e}_{i{j}^{+}}{\sim}^{ind}\ {N}_K\left(0,{\Sigma}_{i{j}^{+}}\right)$. Hence the conditional distribution of the future value given the observations is Gaussian with mean and variance:

$$E\left({Y}_{i{j}^{+}}|{Y}_i={y}_i\right)={X}_{i{j}^{+}}\beta +{C}_{i{j}^{+}}^T{V}_i^{-1}\left({y}_i-{X}_i\beta \right)$$

$$Var\left({Y}_{i{j}^{+}}|{Y}_i={y}_i\right)={V}_{i{j}^{+}}-{C}_{i{j}^{+}}^T{V}_i^{-1}{C}_{i{j}^{+}}$$

For each patient *i*, the predicted probabilities of the major clinical events occurring at ${t}_{i{j}^{+}}$ are given by the Gaussian cumulative conditional distribution function defined above evaluated at the quantalized thresholds *c*_*EF*_, *c*_*RVSP*_, and *c*_*pFVC*_.

### Cross-validated sequential prediction (CVSP) for multivariate longitudinal data

Refitting our multivariate mixed effects model whenever new data are collected is computationally expensive and beyond what is practical in a clinical setting. In addition to having clinical utility, a model must be systematically evaluated and curated over time.

One way to surmount this computational burden and additionally to provide unbiased estimates of prediction error is to use cross-validation in combination with sequential prediction for each patient. That is, we perform 5-fold cross validation by leaving out a randomly selected 20% of the data then refitting the model to produce 5 sets of parameters estimates from the existing data. When a prediction is needed for patient *i*, we use the estimated parameters $\hat{D}$ and ${\hat{\Sigma}}_i$ estimated from the subset of data from which was excluded to calculate *E*(*Y*_*i*(*j* + 1)*k*_| *Y*_*i*_ = *y*_*i*_) and *Var*(*Y*_*i*(*j* + 1)*k*_| *Y*_*i*_ = *y*_*i*_) as well as $\hat{P}\left({E}_{EF,i{j}^{+}}\right),\hat{P}\left({E}_{RVSP,i{j}^{+}}\right),$ and $\hat{P}\left({E}_{pFVC,i{j}^{+}}\right)$. To evaluate prediction error for patient *i* at all his observation times ${t}_{i1},\dots, {t}_{i{n}_i}$ (regardless of which biomarker is measured at each *t*_*ij*_), we sequentially move from the first to last observation. At each *t*_*ij*_, we obtain the prediction of multiple biomarkers at the next observation time *t*_*i*(*j* + 1)_ by calculating *E*(*Y*_*i*(*j* + 1)_| *Y*_*i*_ = *y*_*i*_), where as above *y*_*i*_ is the history of the process prior to *t*_*i*(*j* + 1)_. We will refer to this approach as Cross-validated Sequential Prediction (CVSP). To evaluate the CVSP’s performance, we calculate cross-validated area under the ROC curve (CV-AUC) from 5-fold cross-validation for each of the three events at two levels of severity.

### Checking of joint Gaussian assumption

Although each outcome variable is preprocessed to follow a Gaussian distribution, there is no guarantee that the vector of transformed variables follows a multivariate Gaussian distribution. As our prediction model depends on the joint Gaussian assumption of the random effects and random errors, we propose a method of checking for systematic departures that may affect the performance of prediction by examining the marginal residuals of the model.

The residuals from the linear regression models for person *i*, ${Y}_i-{X}_i\hat{\beta}={Z}_ib_i+{e}_i$ which are approximately Gaussian with mean 0 and covariance matrix *V*_*i*_. We examine the joint Gaussianity of by calculating jointly standardized residuals ${U}_i={\operatorname{diag}}{\left({\hat{V}}_i\right)}^{-\frac{1}{2}}\left({Y}_i-{X}_i\hat{\beta}\right)$ which, under the model, should follow a jointly standardized Gaussian distribution. We examine the Q-Q plots for each measure for all patients where the standardized residuals are plotted against the standard Gaussian and look for obvious departures of the points from the 45 degree line.

### CVSP model specifications

For the fixed effects, the common predictors across all outcomes are age of scleroderma onset, race, gender, skin subtype, and autoantibody status for the 3 most common scleroderma specificities (ACA, RNAPol and Scl-70). To model changes in patients’ health trajectories, we also include a smooth function of time using natural splines with 3 degrees of freedom where internal knots are placed at 10 and 30 years since onset, and boundary knots at 0 and 40 years since onset. The degree of smoothness is guided by the clinicians’ consensus about the typical rate of disease progression in their patient population. The knot locations are chosen nearer to the beginning and end of the total follow-up period (as opposed to equally spaced in time) where more rapid change is anticipated. Note that the set of common variables are allowed to have different coefficients for each measure. Measure-specific regression predictors and coefficients are essential to describe the state of a patient’s scleroderma, as it is known that each clinical subtype is at different risks for organ complications [33]. For example, patients with limited skin type are at higher risk of developing PH but lower risk of developing ILD [34, 35].

**Fig. 3:**
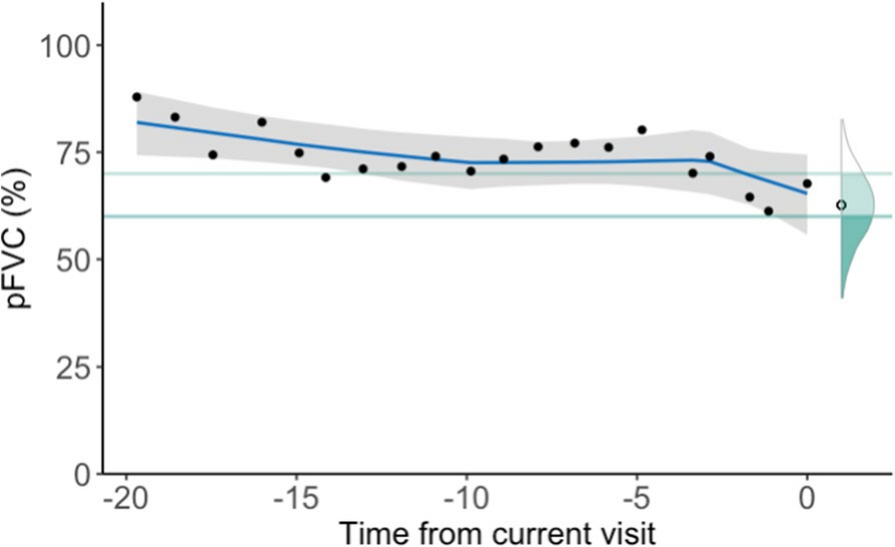
This figure illustrates an individual patient’s trajectory in pFVC over time with the estimated probabilities of crossing two pFVC thresholds 70 and 60 in within the next year. The probabilities are shown by the shaded areas under the curve. The estimated trajectory with uncertainty (blue curve with grey credible intervals) is shown with the actual observed values (black dots). Patient-specific deviation during the 10 and 3 years prior to time 0 is captured in the estimated trajectory

For patient-specific random effects, we fit a random slope and intercept and two linear splines at 3 and 10 years prior to the most recent observation. The same set of predictors are fitted as random effects for all outcome variables. Random effect estimates for the two spline terms represent a person-specific deviation in the linear rate of change during the last 10 and 3 years respectively (See Fig. 3). These terms are introduced to prevent observations early in the disease course from having excessive influence on predicted trends.

### Bayesian hierarchical model framework

We estimate the posterior distribution of the model parameters and functions thereof using Markov Chain Monte Carlo (MCMC) as implemented in the R package MCMCglmm [36]. Gibbs sampling is used to update the parameters of interest, which is possible since conditional distributions are known in our Gaussian outcome case. Details of MCMC sampling in MCMCglmm are in Hadfield, 2021 [37]. We use diffuse conjugate prior distributions for the fixed effects and variances of the random effects and random errors. For fixed effects, we use a diffuse independent Gaussian prior centered at zero with a variance of 10^8^. Diffuse inverse-Wishart priors are placed on the covariance matrices for the random effects and residuals. The degrees of freedom of these prior distributions are chosen to make them as diffuse as possible within their conjugate class. Details regarding the choice of prior distributions are in Supplemental Materials Section A. We examine the convergence of the chains by using the Gelman-Rubin (GR) diagnostic approach [38]. We calculate the potential scale reduction factor (PSRF) of the fixed effects, random effects, and covariance estimates of random effects and conclude the chains have converged for values less than the common threshold 1.1.

### Logistic regression and machine learning prediction models

We compare the performance of our proposed approach implemented using the CVSP against simpler prediction methods including three logistic regression models and a random forest classification model. For predictions using logistic regression, we build a set of models to predict EF, pFVC, and RVSP events, respectively. The three logistic regression models LM1, LM2, and LM3 are defined as follows:

$$LM1: logit\left(\Pr \left({E}_{i\left(j+1\right)k}=1\right)\right)= ns\left({Y}_{ij k},\nu \right)+{Y}_{ij\left(-k\right)}+ common\ covariates$$

$$LM2: logit\left(\Pr \left({E}_{i\left(j+1\right)k}=1\right)\right)= ns\left({Y}_{ij k},\nu \right)+ ns\left({Y}_{i\left(j-1\right)k},\nu \right)+{Y}_{ij\left(-k\right)}+ common\ covariates$$

$$LM3: logit\left(\Pr \left({E}_{i\left(j+1\right)k}=1\right)\right)=\sum_{l<j+1}{E}_{ilk}+ ns\left({Y}_{ij k},\nu \right)+ ns\left({Y}_{i\left(j-1\right)k},\nu \right)+{Y}_{ij\left(-k\right)}+ common\ covariates$$

Here, *logit*(Pr(*E*_*i*(*j* + 1)*k*_ = 1)) is the logarithm of the odds of having an event at time *j* + 1 for patient *i* for measure *k*. As we are not directly modeling the latent trajectory as in the Bayesian hierarchical model, we sequentially add covariates that can summarize past trajectories in multiple measures. *Y*_*ijk*_ and *Y*_*i*(*j* − 1)*k*_ are the most recent and second to the most recent observations of measure *k* to *j* + 1 for patient *i*. We fit a smooth function of *Y*_*ijk*_ and *Y*_*i*(*j* − 1)*k*_ using natural splines with *ν* = 2 degrees of freedom. $\sum_{l<j+1}{E}_{ilk}$ is the count of events in the past for patient *i* before *j*^+^ for measure *k*. The additional information about the past trajectory reflected in *Y*_*i*(*j* − 1)*k*_ and $\sum_{l<j+1}{E}_{ilk}$ may or may not result in improved cross-validated prediction error. We incorporate information from the other three measures by including as predictors patient’s most recent observations prior to *j* + 1. The common covariates are identical to those in the CVSP. Finally, we fit a random forest classification model for each of the binary outcomes [39]. All covariates used in the most comprehensive model LM3 are used as explanatory variables. Further details about the random forest model is in Supplemental Materials Section C.

Unlike the Bayesian multivariate model that flexibly handles missing data in longitudinal outcomes inherently, the logistic regression models and random forest model require an additional step of imputation of the missing covariate values. We are unable to make risk predictions for patients who do not have previous pFVC, pDLCO, EF, or RVSP measurements. Hence, we perform multiple imputation of the missing values using the R package mice [40–42]. To compare the models’ performances to the CVSP’s, we calculate the CV-AUCs for the three logistic regression models from 5-fold cross-validation and AUC computed from out-of-bag (OOB) probability estimates from the random forest model of each event by severity level.

### Checking the calibration of CVSP

We check whether our approach is adequately calibrated by comparing predicted with observed rates of the moderate and severe states for each clinical event in Table 1. We compare the estimated and observed numbers of events within quintiles of the predicted probabilities using a chi-square statistic as a measure of deviation.

## Results

### Data characteristics

We use data from 592 patients who have more than one observation for all pFVC, pDLCO, EF, and RVSP. 6189 pFVC and 5791 pDLCO, 4297 EF, and 3296 RVSP observations are used. EF events are rarer than RVSP events; pFVC events are the most common (Table 2).

**Table 2:** Number of events by severity

	EF < 50	EF < 35	pFVC≤70	pFVC≤60	RVSP≥45	RVSP≥50
Number of events	175	33	2145	1140	414	263

### Checking Gaussian assumption

**Fig. 4:**
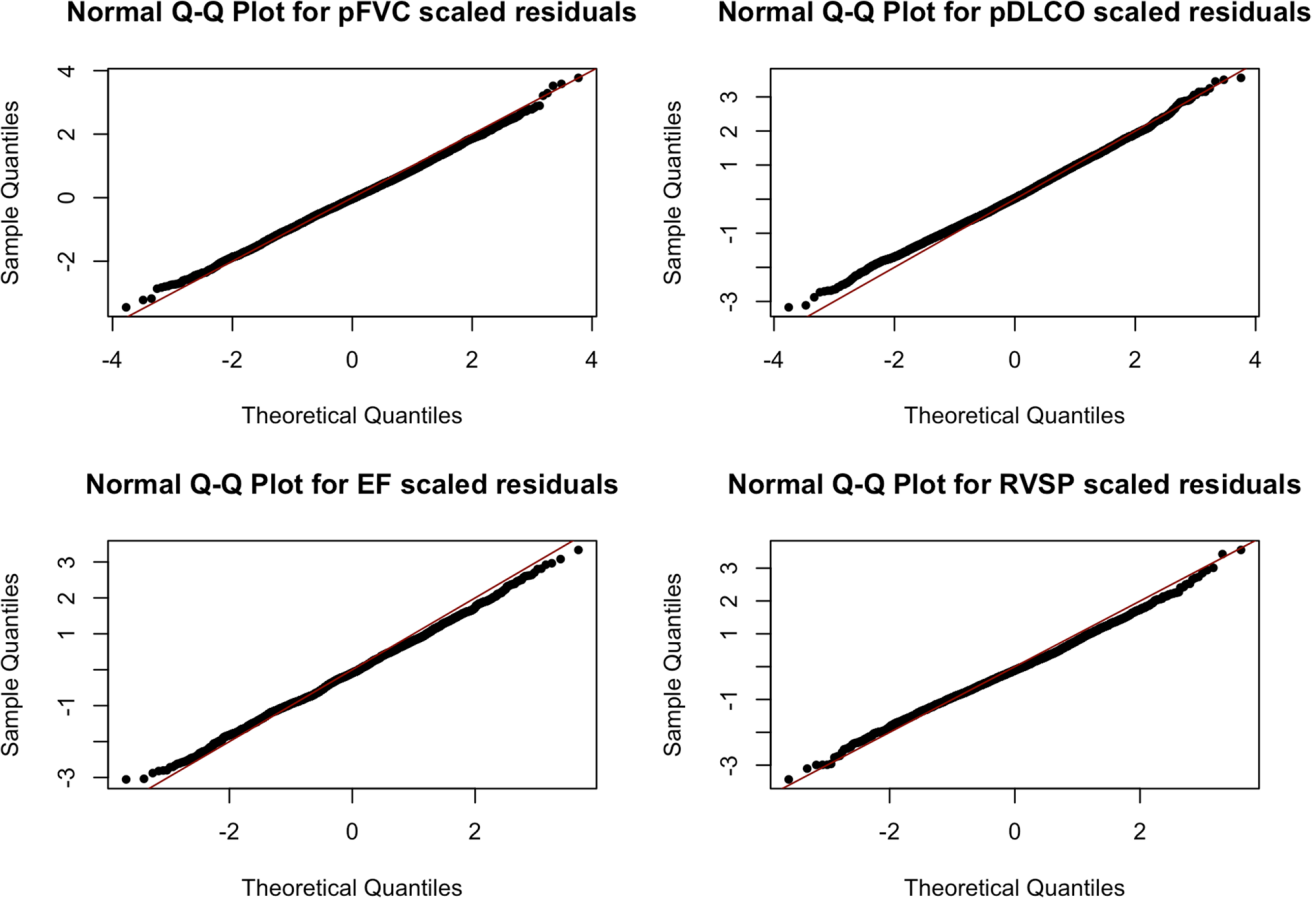
Gaussian Q-Q Plots. The four plots show the sample quantiles of each of the measures in the model against quantiles of standard Gaussian

We would anticipate that the quality of the predictions from our longitudinal model might depend on the joint Gaussian assumption of the transformed biomarkers. Figure 4 shows four plots, one for each of the four measures. In each plot, the Gaussian quantiles on the x-axis and the observed quantiles of the jointly standardized model residuals are on the y-axis. We conclude that there are not substantial departures in the distributions of the scaled residuals from the Gaussian distribution that might compromise the CVSP predictions.

### Convergence diagnostics

We used 50,000 MCMC iterations with burn-in of 2000 and thinning of 10. All PSRFs were below 1.1, indicating that the chains had converged. We also examined trace plots of the chains, which showed no obvious signs of non-convergence. Details regarding convergence diagnostics are included in Supplemental Materials Section B.

### Comparing performances of CVSP, logistic regression, and machine learning methods

The CVSP yields the highest CV-AUC in predicting all events (Table 3). Comparing the events by severity within each of the three measures, the CVSP demonstrates better performance for stricter thresholds (*EF <* 35, *RVSP ≥* 50, and *pFVC ≤* 60). For EF < 35, CV-AUCs from the three logistic regression models range from 0.793 to 0.809 while that of CVSP is 0.854. The random forest model has high precision in predicting EF < 35 (AUC of 0.844) and pFVC ≤ 60 (AUC of 0.944) compared to the logistic regression models but not as high as those of the CVSP. The result suggests that the CVSP may be especially useful in predicting other rare clinical events. For the three logistic regression methods, we observe that sequentially adding covariates that summarize individuals’ history results in improved prediction for all events except for *EF <* 35.

**Table 3:**
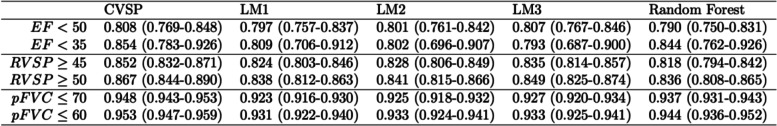
Cross-validated AUC for the CVSP and three logistic regression models (LM1, LM2, LM3), Out-of-bag (OOB) and AUC computed from OOB votes for the random forest method for the three critical events by severity. The associated 95% CI of the AUCs are in parenthesis

All methods show high precision in predicting pFVC events. This implies that the estimated pFVC trajectory for the hierarchical model or even recent pFVC observations coupled with that of pDLCO used as covariates for the other three models are highly predictive of pFVC events. The trend is captured in Fig. 2, where we can observe highly correlated pFVC measurements across time within individuals unlike EF or RVSP. It is likely that jointly modeling pFVC and pDLCO leaving out the cardiac measurements can also produce a highly predictive model.

As expected, the CVSP prediction improves over time as more data are observed. For example, CV-AUC is 0.722 (0.618–0.825) for predicting *EF <* 50 events when no EF measurements are observed. CV-AUC is 0.770 (0.691–0.850) when 1 EF measurement is observed and 0.779 (0.687–0.871) when 2 EF measurements are observed. When there are more than 2 EF measurements, CV-AUC increases to 0.885 (0.834–0.936). In general, the more data a patient has, the better precision is expected. However, even in the case of no previous observations, the CVSP has decent precision, illustrating that patients’ demographic and clincal subtype along with their estimated pFVC, pDLCO, and RVSP trajectories provide reasonable prediction of their future EF trajectory.

### Calibration

In a well calibrated model, the average predicted values should be close to the observed event rates across the range of predicted values. We calculated Chi-square statistics to quantify the size of the deviation between the observed and expected cases in the quintiles of predicted probabilities for each event from all proposed models (Table 4). The CVSP’s calibration is similar to those of LM1 and LM2. LM3 is superior to all others on average and random forest has the poorest calibration.

**Table 4:**
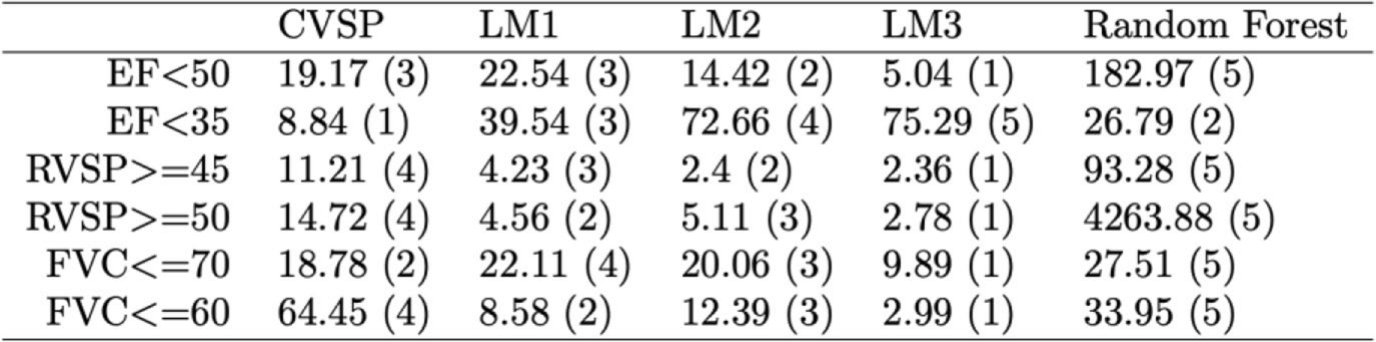
The relative calibrations of the methods. Chi-square statistics (within-row ranks) to quantify the deviations between the predicted and observed numbers of events within quintiles of the predicted probabilities are shown

## Discussion

In the context of predicting critical lung and heart events among scleroderma patients, we have proposed and studied an alternative prediction approach when events are defined in terms of crossing a biomarker threshold level or other function of one or more biomarkers. The common prediction approach is to directly model the binary events or times to events, for example with a logistic or survival model or machine learning alternatives. In this paper, we proposed modeling the multivariate biomarker process itself then calculating the event risks from the fitted model. We demonstrated that our proposed alternate, built from standard linear mixed models and software, produces individualized predictions for scleroderma patients with higher precision and reasonable calibration as compared to the traditional prediction approaches.

Modeling the joint biomarker process directly is feasible because of major advances over the past few decades in statistical modeling and computing for longitudinal data analysis. The linear mixed effects model for multivariate Gaussian data, on which our approach depends, is now commonly estimated using Bayesian Markov Chain Monte Carlo (MCMC) algorithms (JAGS [43], MCMCglmm, Stan [44]) rather than less stable likelihood and restricted likelihood methods (e.g. R package nlme [45], lme4 [46]). MCMC provides inferences about the joint probabilities of threshold crossings with no additional computing or approximations.

But to be practical for real time clinical prediction, the multivariate linear mixed effects model requires two extensions: relaxation of the Gaussian assumption for the biomarkers and simplification of the prediction calculations for new patients using new data. In our motivating scleroderma example, the marginal distributions of RVSP and EF are clearly non-Gaussian. For each biomarker, we replace its observations with the corresponding quantiles for a standard Gaussian variate and transform the thresholds in the same way. The crossing threshold probabilities are unchanged. We further assure that not only the marginal distributions are Gaussian but also that the joint distribution is approximately so, by decorrelating the model residuals and making a Q-Q plot of the uncorrelated values against a standard Gaussian. In our case, the deviations from a Gaussian model to the transformed data are minimal. If they were substantial, the crossing probabilities could be calculated with a different approximating multivariate distribution, for example a multivariate t-distribution with degrees of freedom estimated from the observed decorrelated residuals. In each application, it is important to check the reasonableness of the parametric assumptions and to tailor them as needed.

It would be optimal to refit the biomarker model with each new observation or patient. But fitting a complex model to all the patients’ data takes more computational power than is available in most clinical settings. Ideally, the calculations for a particular patient would be done within the electronic health record system during the patient’s visit. The CVSP algorithm introduced here makes this possible. It combines the information from the population data that is captured in previous model fitting together with the new patient data to make updated predictions. We have successfully implemented the CVSP within our own scleroderma clinic.

That the multivariate biomarker model improves upon the direct predictions in the scleroderma example is likely the result of several of its advantages. First, the multivariate biomarker model is fit to all of the biomarker data, while event prediction models must choose explanatory variables that are simple biomarker summaries like the last value or recent slope. Second, the outcomes in the biomarker model, being continuous, contain substantially more information than the dichotomized event outcomes or times to events. Third, the simple biomarker summaries used as predictors are often measured with non-trivial error. The biomarker models smooth the values across time, reducing the measurement error in the prediction setting. Finally, a multivariate model of the biomarkers naturally handles irregularly observed and missing data as is the case in the motivating scleroderma example. Missing data are effectively imputed from the conditional distribution of the missing values given the observed predictors and outcomes. If the data is missing completely at random or at random, the missingness will not bias these mixed effects model results [47].

In this analysis, we focused on the marginal risk of individual events, but we can easily obtain the joint predicted probabilities of multiple events and estimates of other quantities of interest from the models’ joint posterior distributions.

For scleroderma patients, cardiomyopathy, PH, and ILD are events with high morbidity and mortality. Timely risk predictions are essential because they: (1) warn clinicians of higher risk in need of increased monitoring and interventions; (2) reduce concerns in patients at lower risk. To our knowledge, this work represents the first approach to compute personalized risk estimates for multiple scleroderma complications.

Predictor variable selection was done based upon availability of predictors and prior clinical knowledge. Automated variable selection methods are natural extensions of the methods discussed here. There are other modeling choices such as choosing more informative prior distributions or alternative covariance structure specification for random errors and random effects that can also be tailored to the application at hand. Although we demonstrated our approach with an application to scleroderma, we anticipate it may have broader application to other complex diseases that require multiple measures to monitor progression. Additional case-studies and curation of the resulting predictors are warranted.

## Conclusion

In the context of predicting critical lung and heart events among scleroderma patients, an alternative prediction approach based upon standard multivariate linear mixed effects models of transformed biomarker data is more precise than traditional regression and machine learning methods when events are defined in terms of crossing a biomarker threshold level or other functions of one or more biomarkers. We developed the CVSP algorithm for real-time, clinic-based calculation of an individual’s risk of future major clinical events using information in multiple biomarkers observed at irregular time points for a clinical reference population. Our model has been successfully applied in a scleroderma clinic.

## Supplementary Information

**Additional file 1:.**

## Data Availability

R code is available at https://github.com/jisoo-kim/Predicting-Ssc-Events. The dataset used and analyzed during the current study are available from the corresponding author upon request.

## Abbreviations

ILD: Interstitial lung disease
PAH: Pulmonary arterial hypertension
PH: Pulmonary hypertension
CVSP: Cross-validated sequential prediction
DAG: Directed acyclic graph
EF: Left ventricular ejection fraction
RVSP: Right ventricular systolic pressure
pFVC: Predicted forced vital capacity
pDLCO: Predicted diffusing capacity of carbon monoxide
MCMC: Markov Chain Monte Carlo
CV-AUC: Cross-validated area under the roc curve
GR: Gelman-Rubin
PSRF: Potential scale reduction factor
OOB: Out-of-bag
