# Supplementary material for: Predicting clinical events using Bayesian multivariate linear mixed models with application to scleroderma
Source: BMC Med Res Methodol. 2021 Nov 14;21:249. doi: 10.1186/s12874-021-01439-y (PMC8590788; doi:10.1186/s12874-021-01439-y)
Supplement: Supplementary file 1 — Additional file 1:. [file 12874_2021_1439_MOESM1_ESM.pdf]

Supplemental Materials

A. Prior distribution for the random effects covariance matrix and random error covariance matrix

The prior distribution for the $p \times p$ random effect covariance matrix D is an inverse-Wishart distribution, $D \sim \mathcal{W}^{-1}(\Psi, \nu)$, where Ψ is $p \times p$ positive definite scale matrix, and $\nu > p - 1$ is the prior degrees of freedom. In our application $p = 16$. The inverse-Wishart is the conjugate prior for the random effects variance matrix in the Gaussian linear mixed models. Our strategy is to select Ψ based upon prior clinical expertise about plausible values for the random effects and to specify $\nu = p = 16$, the smallest allowable value, so that $p(D)$ is most diffuse while still guaranteeing D follows a proper inverse-Wishart distribution.

Specifically, for each of the $K = 4$ measures, we choose Ψ such that the range defined by $\pm 2 \times \sqrt{prior\ variance}$ captures about 95% of the plausible range based upon clinical experience. In our application, this corresponds to setting the modes of the prior distribution for random intercept, slope, and two spline terms for each of the standardized measures to be 1, 0.01, 0.01, and 0.01, respectively.

Similarly, we choose a vague prior for the 4×4 random error covariance matrix that follows inverse-Wishart distribution with the smallest degrees of freedom $\nu = 4$. The modes of the distribution are set to be 1 for all four measures.

B. Gelman-Rubin convergence diagnostics

We evaluate convergence of the MCMC chains of fixed effects, random effects, and random effects covariance. We first present histograms of potential scale reduction factors (PSRFs) of the Gelman-Rubin statistic based on 50000 MCMC iterations with burn-in of 2000 and thinning of 10. Figure B.1 shows the distribution of the PSRFs of the fixed effects and Figure B.2 shows those of random effects covariance estimates.

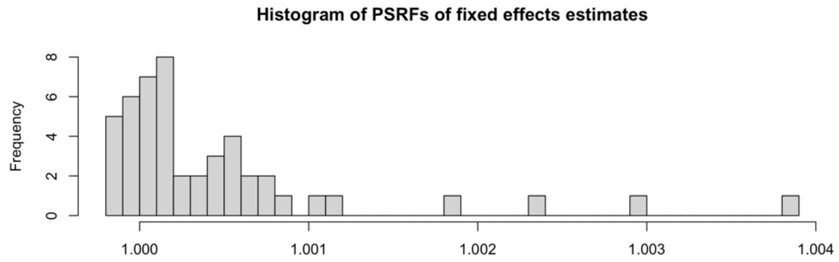

Figure B.1

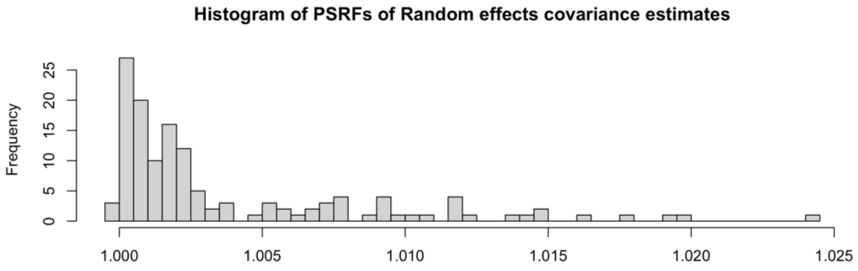

Figure B.2

We also investigated the convergence of the patient specific parameters by calculating and plotting the PSRF for each random effect estimates (Figure B.3.). The PSRFs are close to 1, and no obvious signs of non-convergence are detected.

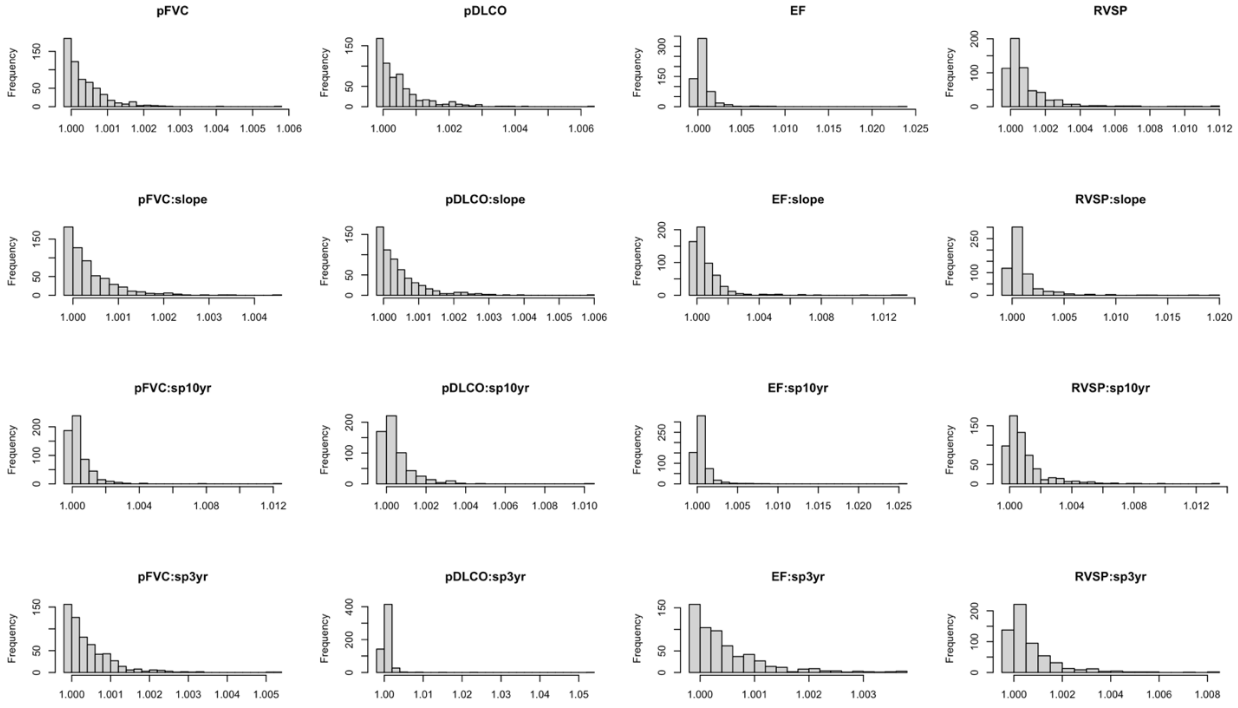

Figure B.3

C. Random Forest classification model

The random forest classification model described in the section "Logistic regression and machine learning prediction models" was fit with r package randomForest [1]. For each of the 6 prediction models, 500 trees are grown, and 3 variables (which is rounded down value of the square root of the number of covariates) are randomly sampled as candidates at each split. The risk of having an event is estimated by out-of-bag (OOB) votes.

References

1. A. Liaw and M. Wiener (2002). Classification and Regression by randomForest. R News 2(3), 18--22.
